# A community-based qualitative study on the experience and understandings of intimate partner violence and HIV vulnerability from the perspectives of female sex workers and male intimate partners in North Karnataka state, India

**DOI:** 10.1186/s12905-018-0554-8

**Published:** 2018-05-11

**Authors:** Andrea K. Blanchard, Sapna G. Nair, Sharon G. Bruce, Chaitanya AIDS Tadegattuva Mahila Sangha, Satyanarayana Ramanaik, Raghavendra Thalinja, Srikanta Murthy, Prakash Javalkar, Priya Pillai, Martine Collumbien, Lori Heise, Shajy Isac, Parinita Bhattacharjee

**Affiliations:** 1 Department of Community Health Sciences, University of Manitoba, 750 Bannatyne Ave, Winnipeg, R3E 0W3 Canada; 2 Karnataka Health Promotion Trust, No 1-4 5th Floor IT Park, Rajajinagar, Bengaluru, 560044 India; 3 Chaitanya Mahila Sangha, Mallamanagar, Mudhol, 587313 India; 4 London School of Hygiene and Tropical Medicine, 15-17 Tavistock Place, London, WC1H 9SH UK; 5 Johns Hopkins Bloomberg School of Public Health, 615 N. Wolfe Street, Baltimore, MD 21205 USA

**Keywords:** India, South Asia, Intimate partner violence, HIV, Prevention programs, Gender, Structural vulnerability, Qualitative, Community-based participatory research

## Abstract

**Background:**

Research has increasingly documented the important role that violence by clients and the police play in exacerbating HIV vulnerability for women in sex work. However few studies have examined violence in the intimate relationships of women in sex work, or drawn on community partnerships to explore the social dynamics involved. A community-based participatory research study was undertaken by community and academic partners leading intimate partner violence (IPV) and HIV prevention programs in Bagalkot district, Karnataka state, India. The purpose was to explore the experience and understandings of intimate partner violence and HIV/AIDS among women in sex work and their intimate partners in Bagalkot that would inform both theory and practice.

**Methods:**

A community-based, interpretive qualitative methodology was used. Data was collected between July and October 2014 through in-depth interviews with 38 participants, including 10 couples, 13 individual female sex workers, and 5 individual male intimate partners. Purposive sampling was done to maximize variation on socio-demographic characteristics. Thematic content analysis was conducted through coding and categorization for each interview question in NVivo 10.0, followed by collaborative analysis to answer the research questions.

**Results:**

The results showed that an array of interrelated, multi-level factors underlay the widespread acceptance and perpetuation of violence and lack of condom use in participants’ intimate relationships. These included individual expectations that justified violence and reflected societal gender norms, compounded by stigma, legal and economic constraints relating to sex work. The results demonstrate that structural vulnerability to IPV and HIV must be addressed not only on the individual and relationship levels to resolve relevant triggers of violence and lack of condom use, but also the societal-level to address gender norms and socio-economic constraints among women in sex work and their partners.

**Conclusion:**

The study contributes to a better understanding on the interplay of individual agency and structural forces at a time when researchers and program planners are increasingly pondering how best to address complex and intersecting social and health issues. Ongoing research should assess the generalizability of the results and the effectiveness of structural interventions aiming to reduce IPV and HIV vulnerability in other contexts.

**Electronic supplementary material:**

The online version of this article (10.1186/s12905-018-0554-8) contains supplementary material, which is available to authorized users.

## Background

In the last few decades, there has been consistent and mounting evidence for the pervasiveness and health consequences of intimate partner violence facing women worldwide. The World Health Organization’s (WHO) definition of intimate partner violence (IPV) is a, “behaviour within an intimate partner relationship that causes physical, sexual or psychological harm, including physical acts of aggression, sexual coercion, psychological abuse or controlling behaviours” [1]. One-third of ever-partnered women globally, and nearly 40% in the South and East Asian regions, reported experiencing physical and/or sexual violence from an intimate partner in their lifetime in 2013, according to the WHO [2]. Research in India and elsewhere has shown that women who sell sex, or female sex workers (FSWs), experience particularly high rates of violence from intimate partners [3–6]. In a study among a representative sample of 1750 FSWs in north Karnataka state, India, 29% of women reported experiencing physical violence from an intimate partner in the last 6 months alone [7].

Research to date has identified a host of important risk factors for partner violence using multivariate analyses. Efforts to extend this analysis to the context of sex work have largely focussed on exploring the impact of client violence on women’s health and wellbeing [8–11]. A few studies have qualitatively explored violence and condom use in intimate relationships among female sex workers, but never with a community-based approach or among both women and their intimate partners [3, 6, 12, 13]. A deeper exploration of how social influences interact to shape the experience of violence among women in sex work and their intimate partners is required if health interventions are to effectively address the underlying processes that heighten IPV and HIV/AIDS vulnerability.

We conducted a qualitative community-based participatory research (CBPR) study to explore the experience and understandings of violence and condom use within intimate relationships among 38 purposively selected participants, including female sex workers and male intimate partners, in Bagalkot district of north Karnataka state, India. This study was part of a mixed method evaluation study of the intimate partner violence prevention program called *Samvedana Plus* being implemented in north Karnataka by the non-governmental organization, Karnataka Health Promotion Trust (KHPT), and their partnering community-based organization (CBO), *Chaitanya AIDS Tadegattuva Mahila Sangha,* in Bagalkot district. In the tradition of community-based participatory research, this study worked with women in sex work as co-learners together with other researchers. The results have been used to adapt the *Samvedana Plus* program over its implementation cycle. In this paper we present our findings, and discuss the implications for IPV and HIV prevention by drawing on a “structural vulnerability” theoretical framework. We aim to contribute to the nascent literature on how an array of social, cultural, economic and political issues interact to perpetuate intimate partner violence and HIV among women in sex work, at a time when population health research and program implementers are increasingly approaching the interwoven social roots of health issues through a complex systems lens [14, 15].

## Methods

### Study design

In response to local interests and needs, the study drew on a community-based participatory research (CBPR), interpretive qualitative methodology [16]. A CBPR approach aims to combine the strengths of community and academic partners to research important issues and inform culturally-appropriate and sustainable interventions [13, 17]. The action-oriented, collaborative approach involved in CBPR aligns well with interpretive forms of inquiry by seeking to understand social phenomena according to those who experience them. This is based on the assumption that “social reality” is necessarily constructed and context-specific, and must therefore be interpreted through meaning-making processes [16, 17]. Hence our methods were collaborative in nature, developing questions able to explore experiences from women’s and men’s perspectives in relevant ways by working closely with community partners, and together undertaking analyses that allowed for multi-dimensional interpretations on how participants conceptualized IPV and low condom use, the root causes, and related ways to address them.

The study was conducted through in-depth collaboration with a Research Committee of women selected by and from the sex worker-led community-based organization *Chaitanya* in Bagalkot. They were involved in the design of the study, oversight of data collection, the selection and training of two female community research investigators (CRIs) to conduct the interviews with female participants, data analysis, interpretation and knowledge translation. A trained male research investigator (RI) from the local region who had been hired by KHPT for previous studies conducted the interviews of male intimate partners (IPs).

### Strategies for enhancing rigour

We aimed to contextualize general guidelines for rigour in terms of pursuing authenticity in both the methods and results of this study. Further details on our approach to pursuing authenticity in CBPR can be found in another article [18]. Briefly, the research team drew on their previous experience and prolonged engagement with each other to develop relevant and sensitive research questions, tools, methods, and modes of analysis that could enhance credibility, or “verisimilitude” between the data and the phenomena they aimed to represent. To ensure dependability of the results, the perspectives of the interviewers and research team, as well as all methodological choices and processes, were recorded by the first author at each stage in an audit trail [19]. We also relied on peer debriefing as we undertook meaning-making workshops with the local CBO and NGO teams after initial coding by the researchers to ensure confirmability or “reproducibility” of the results. Transferability, or the ability to link the results to other contexts, was pursued through our use of purposive sampling that aimed to maximize variation on characteristics reflective of the community socio-demographics, and through detailed description of the research investigators’ observations of the interviews and surrounding context. We also triangulated results on reasons for violence from KHPT’s quantitative behavioural survey among a representative sample of FSWs in the district. In these ways, we aimed to garner trustworthy interpretations on the meaning of the social phenomena under study [16, 19].

The study received ethics approval from St. John’s Medical College in Bangalore, India, the Research Ethics Board at London School of Hygiene and Tropical Medicine, and the Human Research Ethics Board at University of Manitoba.

### Study setting

The study was conducted in the two sub-districts of *Mudhol* and *Jamkhandi* in Bagalkot district in north Karnataka state, India [20]. According to the 2011 Census, the population of Bagalkot was 1.9 million, with almost 70% living rurally. Compared to southern and western Karnataka, literacy and employment rates in 2011 were lower in the northern district of Bagalkot, at 79% and 54% respectively among men, and 58% and 32% respectively among women [20].

*Mudhol* and *Jamkhandi* have been long-term sites for KHPT’s work on HIV prevention, including efforts to develop and strengthen peer-led community-based organizations among FSWs, such as *Chaitanya Mahila Sangha*. This was a response in part to curb the prevalence of HIV in the late 20th century, which was highest in the state and linked to high rates of sex work in the region. This emerged with the historical *Devadasi* tradition that began centuries ago, in which *devadasis* (or ‘servants of the god or goddess’ in *Sanskrit*) were traditionally dedicated or “married” to a god or goddess in the temple, often the goddess *Yellamma*, to perform various religious duties and sometimes sexual services for priests or others [21]. While *Devadasi* women are not permitted to marry legally there are many who have intimate partners in various forms. These may include men who are long-term clients also providing non-monetary or emotional support; men considered husbands, lovers or cohabiting or non-cohabiting non-paying partners; and sometimes pimps with control over their work and finances [5, 22, 23].

Alongside changes in socio-religious practices, and particularly after it was officially banned by the Indian government in the 1980s, *Devadasis* have come to practise more commercialized sex work and now hold a lower socio-economic status than historically [24]. Almost all participants in the current study identified as *Devadasis;* however women who sell sex but who are not traditionally dedicated often adopt the label ‘*Devadasi’* in this region due to some enduring cultural status.

### Participant selection

Participants in this study were purposively selected from KHPT’s intervention records, which contained demographic information on each woman and man enrolled in the *Samvedana Plus* program in 2012 who had reported chronic IPV. This was to maximize variation based on inclusion criteria representing the community from which it was sampled, and found to be related to IPV in past routine monitoring data and according to the Research Committee. These included: those reporting poor condom use in intimate relationships; those with or without alcohol use among either partner; having children or not; both below and above 25 years of age (accepted local cut-off for being younger or older as a sex worker); being married or not; working in sex work only or in other work; and women practising different typologies of sex work (home-based, brothel etc.). The final sample consisted of 38 interviews, including 10 couples (interviewed separately), 13 individual female sex workers, and 5 individual male intimate partners, all of whom provided full participation.

### Research tools

The in-depth interview tools were developed in multiple stages. After the overarching research questions were agreed upon, workshops were held with the community Research Committee to develop theme areas of interest and questions for each. These included: participants’ relationships and supports, problems, hopes and dreams, and expectations in the relationship, followed by experiences, acceptance and methods to address violence and low condom use. The tool was finalized in regional *Kannada*, the local language, based on the input of our community Research Committee, NGO and academic partners in a three-day participatory workshop led by the university researchers with the community research investigators. The CRIs then completed two pilot in-depth interviews and underwent further training with the second author to optimize the data collection process. The interview guides are available as Additional files 1 and 2 for female and male participants respectively.

### Data collection

The female community research investigators went to the villages to meet the potential participants in person and informed them about the study. If they were interested, the CRIs requested their consent. If the potential participant had an intimate partner and she gave her consent to be interviewed, her consent was requested for the male research investigator to meet her intimate partner to seek informed consent for him to be interviewed.

Data collection was completed between July and October 2014. The research investigators conducted semi-structured, one-to-one, in-depth interviews in *Kannada* using audio recorders. These were conducted in the participants’ homes between the interviewer and participant at a single time point for an hour to an hour and a half. Individual in-depth interviews were used for data collection because of the sensitive and exploratory nature of the study questions that aimed to better understand complex issues surrounding norms, beliefs and behaviours [25]. The CRIs also took down detailed field notes based on the training they received, to cover contextual details, non-verbal cues from the participants or circumstances during the interview that may be relevant to the analysis of the transcript. The recordings and field notes were brought back to KHPT’s regional office for transcription and translation by a locally trained translator familiar with the interview guide and data collection processes. The interviews were reviewed for accuracy and completeness in English by the second author prior to analysis, who consulted with the research investigators for clarity and meaning where needed. Further interviews were not collected as the first round of coding revealed recurring patterns on each area of the topic guide, indicating that data saturation was sufficiently achieved.

### Data analysis

All de-identified interviews were imported into NVivo 10.0 using pseudonyms (which are used in the Results), and coded by the first and second authors based on the interview questions and evaluation outcomes, adding new codes or sub-codes when necessary. To prevent selectivity based on outside biases or perspectives, the authors who completed coding met with the male and female research investigators to go over their field notes for each interview and to ascertain what they felt were key findings for each interview question across participants. The CRIs presented these key findings in *Kannada* to the Research Committee and intervention team for further triangulation to assess the important categories. Data was not shared back to the participants because it was felt that this could create issues for maintaining confidentiality around their participation in the study. However we ensured stakeholder input was a central part of the interpretation of results, by relying heavily on a collaborative meaning-making process adapted from participatory methods of card-sorting and flow diagram exercises [25]. For this, the main findings were written onto two sets of cards in *Kannada*, and two groups placed these on separate poster boards in order of proximity around the central themes of violence and condom use, which were then explained and discussed between the groups. The outputs and discussions of the collaborative meaning-making analyses were used to prioritize and interpret the relationships between codes, and thereby to answer the research questions.

## Results

### Intimate partner relationships, supports and expectations

The length of intimate relationships varied from less than one to 30 years in length, with an average of 13 years. Half of the male intimate partners started as a client of the female partner. All but two women had children, and a quarter of these women had children with someone other than their current intimate partner. Only a few women said they saw him more than once a week; most said that it was at least once in a month or two. Table 1 presents the socio-demographic characteristics of the participants.

**Table 1:** Sample by participant socio-demographic characteristics

Socio-demographic characteristic	Category	Female participants	Male participants
Total		23	15
Age	21-29	14	3
	30-39	5	9
	40+	4	3
Religion and caste	Hindu, Scheduled caste	23	4
	Hindu, General caste	0	10
	Muslim	0	1
Employment status	Only sex work	2	NA
	Agricultural or labour work	21	4
	Non-labour work	0	11
Educational level	None	21	4
	Primary	2	3
	Secondary	0	6
	Any higher education	0	2
Marital status	Unmarried	19	2 (1 lived with family, 1 with IP)
	Married	1 (registered marriage with IP)	13 (12 married to other women, 1 had registered marriage with IP)
	Cohabiting with partner	3	0

Social circumstances surrounding intimate relationships were important in shaping the relationship dynamics themselves, and the occurrence of violence and condom use within them. All female respondents were responsible for caring and providing for their children, parents, siblings, or grandparents — a reality that sprung from their inability to marry, as one woman expressed:

*I have shouldered the whole responsibility of the family like a man… I am leading the family by shouldering all the responsibilities even more than a male member. (Sharmistha)*

Meanwhile, the support that male and female partners provided each other fell squarely within common gender role expectations. Women often said their partners were “taking responsibility of”, “maintaining” or “running” her life by providing regular sums of money. Frequently, she also hoped he would pay for clothes or jewellery, her children’s births or marriages, or for building her a house or shop. In return, men widely expected their partners to cook and clean for them, satisfy his sexual desires, maintain fidelity by abandoning sex work, stay at home, and generally be “good” with him.

Not only were gender role expectations important in intimate relationships, but they were likened to a marriage. Women almost always expected his level of affection and support to be equivalent with his wife’s if he were married:

*He treats me similar to how others look after their wives, and he gets me things that I desire, also he stays in our house for a day and the next day he will stay at their home [with his wife]…*

For many women, particularly those in long-term relationships, financial support reflected her partner’s love and commitment, as Kaveri shared:

*He does everything from the bottom of his heart. It is proven in his actions. No one supports either financially or in any other way without having love. He doesn’t come here just to satisfy his lust. He comes here thinking that he has family over here. For that he offers everything.*

The framing of the relationship as “like marriage” was also related to aspirations of social acceptance. This was explained clearly by Sharmistha, who observed that many *Devadasis* were shifting away from sex work in a socio-political context where it is criminalized and blamed for the spread of HIV. As a result many have started to rely more on financial support from intimate partners than clients. The importance of having a male partner to support her, as in a marriage, also strongly derived from women’s hopes to improve their and their children’s futures, as Shanta expressed:

*I have to give a good life to my children; I have to send them to a good family… People should praise me that, even though she is a Devadasi she gave a good education to her children. I have that desire that they should not face the words as ‘her mother is a Devadasi and they too became a Devadasi’. They have to praise me that, she had a lover and he is good with her so he gave a good life to her and her children.*

Hence, the socio-economic and political circumstances that participants faced were important in shaping the cultural expectations that they and their families held for their lovers.

### Experiences of intimate partner violence

Women frequently reported emotional or verbal violence and controlling behaviour, as well as some cases of sexual coercion, though these were not always interpreted as “violence”. Respondents most commonly reported physical violence in terms of slapping or hitting, which was often mutual and considered unserious. Usually longer term partners said they had adjusted or matured, leading to less fights or violence. In only a few cases women said that physical violence occurred routinely, with reported incidents of a bloody head or pulled hair. Some men said there was no violence that occurred, even when the female partner reported it, while other men said there were occasional fights where he had slapped or hit her.

### Understandings of intimate partner violence and condom use

#### Gender role expectations and “mistakes”

Our analysis revealed that unmet expectations of their partners were most commonly the triggers of fights or beating. Conflicts often occurred when men could not fulfil women’s expectation that their partner should be present and provide financial support or material goods, as Sangita responded:

*We quarrel while giving or taking a few things. I shout at him if he delays his arrival after fixing the time for going to the market or elsewhere.*

A major reason why violence was said to occur was when the female partner contravened the gender role expectations her male partner had for her, which were universally classified as “mistakes”, as Ravi explained:

*Any man will get angry if women do ‘mistakes’. He will not get angry if she cooks and serves properly and she obeys him.*

One woman shared how conflict arose when her partner expected to have intercourse more than she wished:

*He wants to have sex every other day. I cannot do it frequently. I say to him to come, be with me, spend some time with me and go. Then he argues with me.*

Women also said violence was triggered if they disregarded men’s expectations around modesty, as Sangita stated:

*They [partners] doubt women, they question, ‘why does she get ready beautifully, why does she go out?’ They force women to stay at home, they restrict their mobility, so [then] violence takes place.*

#### Trust, fidelity and sex work

Among the most common of the unmet expectations said to cause violence were trust and fidelity between partners. In a few cases, a woman fought with her partner if he showed affection or talked to others, as Ajay related:

*If my phone remains busy when she was calling, she doubts me saying that I am in another relationship with someone. I become upset then. She blames me like this. I smack her then. She doesn’t eat anything for couple of days, she keeps quiet. After a couple of days, we again become normal.*

Distrust from the man’s side related most greatly to his disapproval of her engagement in sex work, which he expected to stop when he was supporting her, as Rani’s experience demonstrates:

*He shouted at me saying that I was not at all trustworthy to him despite the fact that he had done so much for me and I had looked for other clients… It has been happening always. He is catching me red-handed every now and then. He scolds me, beats me, I cry. He makes up with me… How can his love towards me allow him to avoid me completely?*

A number of women indicated the distress and fear they experienced when their partners were monitoring them through friends or recording their phone calls. Almost all men said they believed that their partner had stopped sex work, but many women continued covertly for financial reasons. There was also noticeable stigma when men and women both discussed how sex work was viewed, using terms like “dark side”, “vulgar work”, or “whore”. Hence, stigma around sex work converged with suspicions around infidelity to produce emotional turmoil and violence within intimate relationships.

#### Condom use negotiation

Suspicions of infidelity also related to women’s requests to use condoms, which could lead to disagreements and violence. Though partners often started as clients, condoms were discontinued once they considered it an exclusive relationship or rejected outright, as one woman reported:

*He said, ‘Hey, don’t you know to whom it [condom] is given [for sex work]? Throw that away, why are you giving that to me? I am not that kind of person’. He was shouting like this. He was not using the condoms… Once he came to beat me when I asked him to use condoms; I think I tried couple of times. He never listened to me.*

Indu expressed how condoms symbolized HIV prevention, and by extension, non-exclusive or client sexual relationships:

*They [lovers] question us on whether [we think] they have any diseases like AIDS and why should we want them to use the condoms. They show their anger if we force them, and say that if we force them they would leave us… What can I do now, if I insist that my lover use condoms he threatens me like that. I can insist that my clients use condoms when I do sex work secretly, but if we force the lover he would leave me; how can I lead my life then?*

Thus lack of condom use was due to a combination of expectations for fidelity, their perceived association with sex work and HIV, as well as women’s fears of violence or losing the man’s presence and support.

#### Acceptance of violence and its perpetuation

Another major issue said to contribute to IPV was the acceptance of violence by women, their partners and wider community; however in some accounts non-acceptance of violence was also seen to cause violence. Overall, violence was seen to be acceptable among both men and women if the female partner had made a “mistake” while the partner was supporting her, as Nandhini stated:

*Because men will give everything for the household, so they have that right to beat us, but they should beat us only if we do the ‘mistake’.*

This appeared to be rooted in normative expectations of a marital relationship, which Ravi expressed:

*She should not have any contact with other men, because I have taken the responsibility of her life. So I will beat her if she commits ‘mistakes’. I will be like a husband with [lover’s name] as how you [interviewer] are with your wife.*

Further, women and men stated how violence was acceptable because it was a sign of love, as one intimate partner stated:

*When we fight each other, I show more love towards her… Yes sir, there is a saying, ‘Where there is love, there is a fight’.*

One woman shared how others also told her to accept violence if he was supporting her, and that she viewed it as a sign of love:

*Interviewer: Okay when you fight like this, do other women tell you that you should accept it?*

*Respondent: Yes, they tell that I should accept though he beats me, hits me, as he looks after everything for me. They told me to adjust with him… I feel as if I was beaten by my husband, no one else.*

*I: You believe that he is like your husband though he had beaten you and you don’t mind at all?*

*R: Yes, because when he becomes angry, he hits me, blames me, but I lead my life believing that he is like my husband and no one else. I think as if he hits me because he loves me.*

Conversely, a handful of women said that they did not see violence as correct but accepted it for pragmatic reasons such as fear that he would leave, or shame to be exposed in public, as Rani stated:

*When we accept the man whom we love we have to accept everything from him, he might send us out, we have to accept beating from him and we have to go to him when he calls us on the bed. For that sake they keep us.*

Further, a couple women shared that if women don’t accept violence it will lead to more fights:

*Those who do not accept violence, they become a victim [of her partner] and those who accept violence they will be happy after compromise. (Parvati)*

*Interviewer: What happens if you won’t accept violence from your lover?*

*Respondent: That will be the basic cause for a fight. (Sita)*

In the male partners’ views, many felt violence was only justified if to correct her behaviour, as Ravi said:

*It is correct to beat when they commit the mistake. If we beat them without any reason or without them doing any wrong, my hands will be cut-off; I will get wounds on my hand.*

Ajay also discussed that despite its role to correct behaviour, violence was seen as undesirable if frequent and unjustified:

*If we come and beat the wives every day, it doesn’t look good. We will be in the bad books of our neighbours too… Life shouldn’t be like ‘dosa [flat bread] at everyone’s home has holes’.*

Though less common, a few women felt that they should never accept violence. A distinctive account was given by Kaveri, a long-time CBO peer educator, who said that violence was fundamentally wrong:

*[We should not accept it] because we treat them very well bearing in mind that they are like a husband. They should also respect us and treat us in a dignified manner. Do you think we have kept them to behave like that? We don’t. That is the reason.*

In a similar way, Rani’s lack of acceptance for violence was fuelled by her belief that she needed to remain independent as a *Devadasi* woman:

*We have been offering them everything; they take care of us by arranging everything for our needs. That is it. Why should we bend? Whatever it may be, they cannot take care of us as same as they do for their wives. Though anything goes wrong, we won’t get the status of wife. Everyone including our lover treats us as ‘whore’ on one or the other day. In such conditions, why should we get scared about them? Now he has love and affection towards me, who knows, what would happen tomorrow?*

Thus, the women least likely to accept violence were those who either felt prepared for him to leave, or were confident enough that he would not.

#### Methods to address intimate partner violence

The participants’ levels of acceptance aligned with their recommendations for dealing with violence. As Sita explained, if the mistake was from the woman’s side then she must change her own behaviour. However, if she had not made a mistake then her family would come to intervene:

*If the mistake is from the woman’s side it will stop [she will adjust and the dispute will end]; if the mistake is from his side, her family member’s will not become quiet.*

One male intimate partner shared a revealing account of the challenges that *Devadasis* faced in dealing with violence as unofficial partners:

*If there is violence or a fight between a wife and a husband, we can go to them and counsel them not to do that. It is our duty as an elder, and it is correct to say so in such a relationship. However, no one says anything to lovers.*

Similarly, Indu told the interviewer that the neighbours did not lend support because it was considered an illegitimate relationship:

*When we quarrel like that, we don’t let anyone of the neighbours come to know this because they start undervaluing us, saying that we are not wife and husband and we are quarrelling like this as we are in an extra-marital affair.*

In this context, Kaveri expressed that the power to deal with violence had to come through collective strength within the *Devadasi* community:

*We have all combined together. The life of our women is like that only, everyone sees us contemptibly. So we are strong enough as a group. Don’t allow any violence to take place because it hurts everyone if any of us is attacked. So we don’t get adjusted with everything. We don’t like violence from anyone on us.*

A few women likewise received assistance from the CBO helpline, information on the Domestic Violence Act or advocacy from peer workers for preventing and dealing with violence.

## Discussion

Our community-based qualitative study to explore violence and condom use in the intimate relationships of women in sex work sheds light on the complex linkages between vulnerability to IPV and HIV, and broader factors including societal gender norms, expectations and unequal power relations, as well as socio-economic constraints, stigma and illegality surrounding sex work. Many of these echo the factors that have been found to be quantitatively associated with IPV and HIV risk among women generally, though the strength and mechanism of effects surely vary by context [2, 3, 5, 6, 9, 26–29].

Although the inclusion of couples has been rare in past research on IPV and particularly among women in sex work, taking this approach shed new light on the norms and expectations related to condom use and violence held more broadly in society by virtue of being referenced, though often challenged, in intimate relationships. The exploration of the unique situation of *Devadasi* women, who are most often unable to marry and primary breadwinners in their families, likewise helped to reveal what was considered “normal” or “ideal” behaviours for men and women in relationships. Importantly in this study and similar to others, violence and lack of condom use stemmed greatly from the challenges that both intimate partners faced in reconciling her vocation in sex work with the desire to assume socially-accepted gender roles for exclusive, even marital, relationships [6, 28, 30–32]. Participants’ expectations closely echoed others’ findings on gender roles and norms in marital relationships, including the role of men as providers and women as modest, care-taking partners that have been related to domestic violence elsewhere in India [33–36]. The ascription to marital relationships was also demonstrated when some male and female respondents in our study explained that violence was considered a sign of love, because it reflected that the man cared enough to discipline his partner when she made mistakes as one would in a marriage. Similarly in Kapadia-Kundu et al.’s study among young married women, their husbands and mothers-in-law in south India, women’s “mistakes” around gender role expectations, particularly care-taking, modesty and sex, were seen as the primary causes that justified domestic violence against them [30]. Such resonance in the associations between gender roles and violence between study contexts contributes to Bottorff and colleague’s call for improved understandings not only in terms of individuals’ interactions, but how these speak to meso- and macro-level representations of gender relations in society, and their influences on health [37].

Increasingly researchers have aimed to move beyond individual-level frameworks and a focus on immediate “risk factors” towards understanding socio-structural influences on health. To do this, some have started to apply frameworks of “structural vulnerability” that are derived from theories of structural violence [38–40]. Drawing on the concept of “structural vulnerability” encourages a broader appreciation of how one’s experience of violence is shaped not only at the individual or interpersonal level, but also through the influence of community and societal factors that involve relations of power [28, 38]. In this study, socio-economic, cultural, political and legal issues that have been independently associated with violence in quantitative studies appeared to interact with one another and with individual participants’ experiences to shape the degree of “structural vulnerability” to IPV and HIV that they faced [41, 42]. First, male intimate partner’s financial support was crucial for women who experienced stigma and being low caste as sex workers, within cultural and legal structures preventing them from marrying and criminalizing them, and the economic challenges of supporting their families as unmarried women. Yet women in sex work reported having more difficulty fulfilling the gender role expectations for women and thus were more likely to make “mistakes”, particularly by engaging in sex work. These factors also made it more likely for them to comply with the intimate partner and stay with them, in spite of violence or inability to negotiate condoms. This was based on a reportedly widespread societal acceptance of violence if women made “mistakes”, as well as the association of condoms with non-exclusive and client relationships. The latter may have been inadvertently fuelled by past HIV prevention messages that focussed solely on condom use with clients, as others have found [5, 28, 32]. Finally, female respondents felt that society especially blamed them for experiencing violence because the same legitimacy was not attributed to non-formal partnerships as to marital relationships. These results help explain why the categories reported as most contributing to IPV in KHPT’s representative quantitative survey of FSWs were their “engagement in sex work”, “financial constraint”, followed by their “subordinate position in society” [43].

Our findings on the multi-levelled issues shaping “structural vulnerability” to IPV and HIV confirm that continued reliance on behaviour change models to help women avoid “mistakes” or reduce their conditional acceptance of violence are insufficient to address, and may even exacerbate, violence and HIV [38, 40, 44]. Here it is relevant to draw on the growing application of theories on the interrelationship of social structures and individual agency when trying to understand social influences on health [45, 46]. Connell’s relational theory provides a pertinent example in which gender as a social structure, which others have described as both rules and resources, can be understood as shaping as well as being shaped by individuals’ practices (e.g. IPV) through their position in interpersonal power relations and social conditions [45, 47, 48]. Related conclusions have been made by Jewkes and colleagues from their research on violence and gender, who state that, “[m]asculinities are embodied and reproduced across the social ecology, and thus [violence] interventions must seek changes at multiple levels” [49]. Our findings suggest that future research should conceive of health issues like IPV not only in relation to multiple levels of factors, as outlined in important socio-ecological models for IPV [50], but also the interplay across levels, to better conceptualize how socio-structural factors influence and are influenced by individual and collective practices [51].

Effective structural interventions to address IPV vulnerability will likely be those that partner with communities to address pre-existing arrangements of power within the social context; analogous approaches have already been found to be effective for HIV prevention among FSWs [9, 52, 53]. Through a “structural vulnerability” lens, the involvement of male partners in conflict and anger management must be coupled with work on societal-level platforms to cause sufficient change in structural rules such as gender norms that promote acceptance of violence [54, 55]. Further, such structural interventions would continue to work to augment women’s access to important material and social resources through access to entitlements, advocacy against stigma and discrimination, economic assistance, and just enactment of laws [54, 56].

### Limitations

We aimed to maximize representation in our purposive sample, but selection bias could have arisen if those who consented shared milder cases or were less afraid to discuss violence. However the original intervention database from which the sample was derived only included couples known to have reported IPV and low condom use previously. There was likely some social desirability bias or non-disclosure in the accounts, particularly around the extent of conflict and condom use. Community research investigators’ perceptions on each respondent’s level of comfort and openness in the interviews likely aided in ensuring trustworthiness in these areas. Though the interviewers had to develop their skills in a short period, it was clear that after the first few interviews they became much more in-depth with support from the research team.

## Conclusions

Our community-based research study among female sex workers and their male intimate partners in Karnataka exposed the ways that gender role expectations between intimate partners, by virtue of reflecting those widely-held for marital relationships, can lead to IPV and low condom use. Such interpersonal issues were further compounded by the complex interaction of broader factors like stigma and socio-economic constraints, making it more difficult for FSWs to fulfil their partners’ expectations, and thereby heightened their structural vulnerability to HIV and IPV. Our results support the potential for sensitively involving not only women but men and community members in violence research and programs, as their views and actions were not isolated, but referenced wider perspectives on gender relations and violence. Future research to understand how complex and multi-levelled issues shape structural vulnerability to health issues by joining forces between community and academic partners will help to better bridge knowledge and action, and thereby inform relevant and effective prevention programs in other contexts.

## Additional files

Additional file 1: Interview Guide with Female Participants. (PDF 269 kb)

Additional file 2: Interview Guide with Male Participants. (PDF 405 kb)

## Abbreviations

CBO: Community-based organization
CBPR: Community-based participatory research
CRI: Community research investigator
FSW: Female sex worker
HIV: Human immunodeficiency virus
IPV: Intimate partner violence
KHPT: Karnataka Health Promotion Trust
