# Supplementary material for: A community-based qualitative study on the experience and understandings of intimate partner violence and HIV vulnerability from the perspectives of female sex workers and male intimate partners in North Karnataka state, India
Source: BMC Womens Health. 2018 May 11;18:66. doi: 10.1186/s12905-018-0554-8 (PMC5948786; doi:10.1186/s12905-018-0554-8)
Supplement: Supplementary file 1 — Interview Guide with Female Participants. (PDF 269 kb) [file 12905_2018_554_MOESM1_ESM.pdf]

Interview Guide with Female Participants		
**Topics**	**Sub-topics**	**Questions and probes**
Important relationships in our lives	Different people in life<br><br>Support: give and get	• Who is in your daily life? Tell me all people you relate to on a regular basis... Who else? (If people are clearly missing, probe: Your lover/intimate partner (IP)? Peers?) *(Draw relationship map around a circle)*<br>• *For each individual around the circle:* What do you give and what do you get, in terms of support from them (practical, emotional/love, money)?
What about me?	Perceptions of self<br><br>Perceptions by others	• What do you like about yourself? What are your good qualities?<br>• What qualities do you see in others you would also like to have? Which one would you like most? Do you believe it is possible for you to change that?<br>• *For each individual around the circle*: What do they like/not like most about you? Why/why not?<br>• Among these people, who knows you earn money by selling sex?
Problems	Most important problems	• What are the problems you currently face in your life? Any others?<br>• Tell me more about your main current problem? What happened? What did you do?
Intimate relationships	Expectations vs. reality in IP / FSW relationships<br><br>Acceptance of/by IP	• Tell me a bit more about your IP: what do you like most about him?<br>• How do you express your love to him? How does he express his love to you?<br>• What are your expectations from him? Which ones does he not fulfill?<br>• What are his expectations from you? Which of those can you not fulfil?<br>• How connected or accepted is he by other important people in your life?
Violence in the IP relationship (*himsa*)	Current/recent experience with violence<br><br>Ability to deal with violence	• What are the most important disagreements in your relationship?<br>• What type of abusive or violent behaviour do you experience? Probe: Emotional (threats/controlling behaviour) /physical /sexual violence?<br>• What are the triggers for violence? Probe: What are different things that set it off?<br>• Tell me about your most recent experience of violence? What happened? What set it off?<br>• What did you do? What else? Probe: From whom did you seek support? Anyone else?

	Norms about violence	• Are there ways you can avert violence from your IP? Probe: Do you have any strategies? What do you do?<br>• How confident do you feel that you can avoid violence from your IP in future?<br>• Do most FSWs in the community accept violence from IPs? What happens to those that do not accept violence?<br>• Do important people in your life think you should accept violence from your IP? Who does not?
Condom use in intimate relationships	Condom use	• I want to ask some questions about condoms: do you think other FSWs like you use condoms with their IP? Out of 10, how many use condoms?<br>• Do other FSW women think you should use condoms with your IP?<br>• When do you use condoms with your IP?<br>• What happens if you insist on using condoms with your IP?
Intervention	Exposure to intervention<br><br><br>Knowledge on violence laws	• When experiencing violence, who can women approach?<br>• Has anyone approached you about dealing with violence? What support did you get? Probe: Anything specific on violence with lovers?<br>• What was new? What was helpful?<br>• Have you heard about laws to stop men using violence against women? Where did you hear about this? Tell me more…<br><br>*... To end:*<br>• What are the changes you want to make to feel happy and secure?<br><br>*Debriefing: Thank you for taking the time to talk with me. How did you find the interview? Do you have any questions?*
