# Supplementary material for: A community-based qualitative study on the experience and understandings of intimate partner violence and HIV vulnerability from the perspectives of female sex workers and male intimate partners in North Karnataka state, India
Source: BMC Womens Health. 2018 May 11;18:66. doi: 10.1186/s12905-018-0554-8 (PMC5948786; doi:10.1186/s12905-018-0554-8)
Supplement: Supplementary file 2 — Interview Guide with Male Participants. (PDF 405 kb) [file 12905_2018_554_MOESM2_ESM.pdf]

Interview Guide with Male Participants		
**Topics**	**Sub-topics**	**Questions and probes**
Important relationships in our lives	Different people in life<br><br>Support: give and get	• Who is in your daily life? Tell me all people you relate to on a regular basis... Who else? (If clearly missing, probe: Your lover/intimate partner (IP)? Peers?) *(Draw relationship map around a circle)*<br>• *For each individual around the circle:* What do you give and what do you get, in terms of support (practical, emotional/love, money)?
Intimate relationships	Expectations vs. reality in IP / FSW relationships:<br><br>Acceptance of/by IP<br><br>Roles and duties in the relationship	• Could you describe your relation with your lover a little more? What do you like or dislike about her? How do you contribute back to her life? What responsibilities do you share with her?<br>• How do you express your love to her? How does she express her love to you?<br>• What are your expectations from her? Which ones does she not fulfill?<br>• What are her expectations from you? Which of those can you not fulfil?<br>• Could you describe your relationship with her family? How do you adjust with her family members?<br>• How connected/accepted is she to other important people in your life?<br>• What duties and roles as a man should one have in a close relationship? What duties/roles as a woman should a female partner have? What should (or does) happen if one does not fulfil their duties? Can you give an example when this might have occurred in your own relationship?<br>• What are the most important disagreements in your relationship?
Violence in the relationship	Violence in general / Norms about violence<br><br>Violence in the relationship	• Violence in domestic relationships is an issue that people are talking about. Do you think this is common? How would you describe “violence” in relationships?<br>• Do you think it is ever okay to hit/beat a woman? Under what situations?<br>• Is it acceptable for men in your community to beat women? If yes, under what situations?<br>• What do you think will happen if one does not use violence against women, wives or lovers?<br>• Particularly in your own relation with your lover, what types of disagreements do you have? What happens in those circumstances? What leads to this situation of violence? (Probe: money, jealousy, alcohol or others?)<br>• What type of violence (beating, sexual, verbal abuse)? What type is more common? When does this happen?

		• What do you feel after this happens? (Probe if express remorse: What are the solutions for dealing with this? Do you feel satisfied with what you do to deal with violence? Why or why not?)
Condom use in intimate relationships	Condom use	• I want to ask some question about condoms: Do you think other men in your community use condoms with their lovers? Out of 10 of them, how many do you think use condoms?<br>• Do other men think you should use condoms with your lover?<br>• When do you use condoms with your lover?<br>• What happens if she insists on using condoms with you?
Intervention	Exposure to intervention<br><br>Knowledge on violence laws	• Has anyone approached you with information about violence? What did you get to know? Probe: Anything specific on violence with lovers?<br>• What was new? What was helpful?<br>• Have you heard about laws to stop men using violence against women? Where did you hear about this? Tell me more…<br><br>*Debriefing: Thank you for taking the time to talk with me. How did you find the interview? Do you have any questions?*
